# Atherogenic index of plasma for non-diabetic, coronary artery disease patients after percutaneous coronary intervention: a prospective study of the long-term outcomes in China

**DOI:** 10.1186/s12933-022-01459-y

**Published:** 2022-02-22

**Authors:** Yitian Zheng, Chen Li, Jie Yang, Samuel Seery, Yu Qi, Wenyao Wang, Kuo Zhang, Chunli Shao, Yi-Da Tang

**Affiliations:** 1 Department of Cardiology, State Key Laboratory of Cardiovascular Disease, Fuwai Hospital, National Center for Cardiovascular Diseases, Chinese Academy of Medical Sciences and Peking Union Medical College, Beijing, China; 2 Graduate School of Peking Union Medical College, Chinese Academy of Medical Sciences and Peking Union Medical College, Beijing, China; 3 School of Humanities and Social Sciences, Chinese Academy of Medical Science & Peking Union Medical College, Beijing, China; 4 Faculty of Health and Medicine, Division of Health Research, Lancaster University, Lancaster, UK; 5 Department of Cardiology and Institute of Vascular Medicine, Peking University Third Hospital; NHC Key Laboratory of Cardiovascular Molecular Biology and Regulatory Peptides; Key Laboratory of Molecular Cardiovascular Science, Ministry of Education; Beijing Key Laboratory of Cardiovascular Receptors Research, Beijing, 100191 China

**Keywords:** AIP index, Coronary artery disease, Percutaneous intervention, Non-diabetic, Metabolic syndrome

## Abstract

**Background:**

Non-diabetic coronary artery disease (CAD) patients are thought to encounter metabolic dysfunction and while these changes may be imperceptible to the patient they probably influence outcomes. At present, there is no system to support patients *sensing* these subtle changes, nor is there an established model for prognoses. The Atherogenic Index of Plasma (AIP) index has already proven useful for atherosclerosis although further research is needed, especially for those without hyperglycemia.

**Methods:**

This is a prospective study of 5538 non-diabetic CAD patients who had received percutaneous coronary intervention (PCI). Participants were assigned to one of three groups according to their AIP index. High AIP index cases were then compared to low index patients according to major adverse cardiac events (MACE). Restricted cubic spline (RCS) analysis was also conducted to investigate interrelations between AIP index levels and hazard ratios (HR) for MACEs.

**Results:**

Patients with a high AIP index encountered metabolic dysfunction compared to those with a low AIP index i.e., higher Body Mass Index (BMI), Total Cholesterol (TC), Triglycerides (TG), and uric acid as well as lower HDL-C. Each of the aforementioned interrelations were significant with *p* values of less than 0.001. There was also a significant increase in the number of MACEs in the high AIP index group compared to the low AIP index group (HR: 1.37, 95% CI 1.04–1.81; *p* = 0.025). A J-shaped RCS curve highlighted a change in the HR after the 0.18 juncture (HR per SD: 1.20, 95% CI 0.96–1.50). Further subgroup analysis supported the main findings, all with HRs greater than one.

**Conclusion:**

The AIP index could be used in prognostics for non-diabetic CAD patients 2 years after PCI. The relationship between hazard ratio and the AIP index appears to be J-shaped. Although, further multi-center studies designed for non-diabetic patients with potential metabolic dysfunction should be conducted to determine the value of the AIP index.

## Background

Coronary artery disease (CAD) is the world’s leading cause of death [1], with individual prognosis dependent upon multiple factors, such as diabetes or the existence of metabolic syndrome [2–4]. While medication and revascularization therapeutics have greatly improved prognosis, there remains a number of limitations which must be addressed. So, while percutaneous coronary intervention (PCI) is the commonly used revascularization intervention for CAD, little is known about how metabolic disorders influence the efficacy of this intervention. We know, Diabetic Mellitus (DM) is one of the dominant conditions and affects metabolic functions in those with CAD [5]. And, multiple indexes have been used in prognostics for DM patients who comorbidly suffer CAD [6, 7]. However, for CAD patients who do not have comorbid DM, metabolic disorders, such as insulin resistance and metabolic syndrome are largely ignored. This creates a gap in our understanding and one which ought to be investigated to gain a clearer picture of interactions.

There are several prospective interventions for CAD although a decision to utilize a specific approach tends to be used only in specific cases or when there appears to be multiple vessel disease. For example, evidence suggests that Coronary Artery Bypass Grafting (CABG) may be superior in certain instances [8, 9]; however, CABG is also a more invasive procedure. Therefore, through the shared decision-making processes between patient and practitioner, patients tend to choose percutaneous coronary intervention (PCI). These interventions comprise a number of technologies which are capable of relieving coronary narrowing. PCI (also known as coronary angioplasty) broadly consists of rotational, directional or extractional atherectomy or laser angioplasty. Therefore, there are a number of technological differences which may also influence outcomes. While global health research suggests that over one million PCI administered each year [10] to CAD patients, there are differences between the sexes and ethnicities which ought to be explored. For example, researchers have found disparities between African Americans and Caucasian Americans and European Caucasians with CAD after PCI [11–13]. So clearly, we need to gain insight into PCI related technological differences and to understand differences between ethnicities in order to individualize revascularization strategies. However, there is also a need to utilize a standard assessment index for consistency.

The Atherogenic Index of Plasma (hereafter referred to as the AIP index) has been used to ascertain atherogenic dyslipidemia, and is calculated as follows log10 [TG/HDL-C] [14]. The AIP index has proven to be a reliable predictor for CAD patients with type 2 diabetes and is closely associated with lipoprotein particle size, which reflects atherogenic dyslipidemia characteristics [14, 15]. Previous studies have also reported a close relationship between the AIP index and insulin resistance, diabetes mellitus, and metabolic syndrome [15–17]. In two recent studies, researchers reported the predictive value of AIP index and Triglyceride-Glucose index (TyG index) in those with acute coronary syndrome (ACS) and diabetes [6, 7]. These indices could also be used for non-diabetic CAD patient prognosis, although previous research suggests that the TyG index does not accurately predict outcomes for non-diabetic patients after PCI [18]. Therefore, research should prioritize an analysis of the AIP index in combination with Triglyceride analysis, and specifically HDL-C, which is thought to have a “U-shaped” relationship with risk of cardiovascular disease [19]. Using these as the standard indices for non-diabetic CAD patients after PCI may provide useful insights for clinical practice.

## Methods

### Study design

**Fig. 1:**
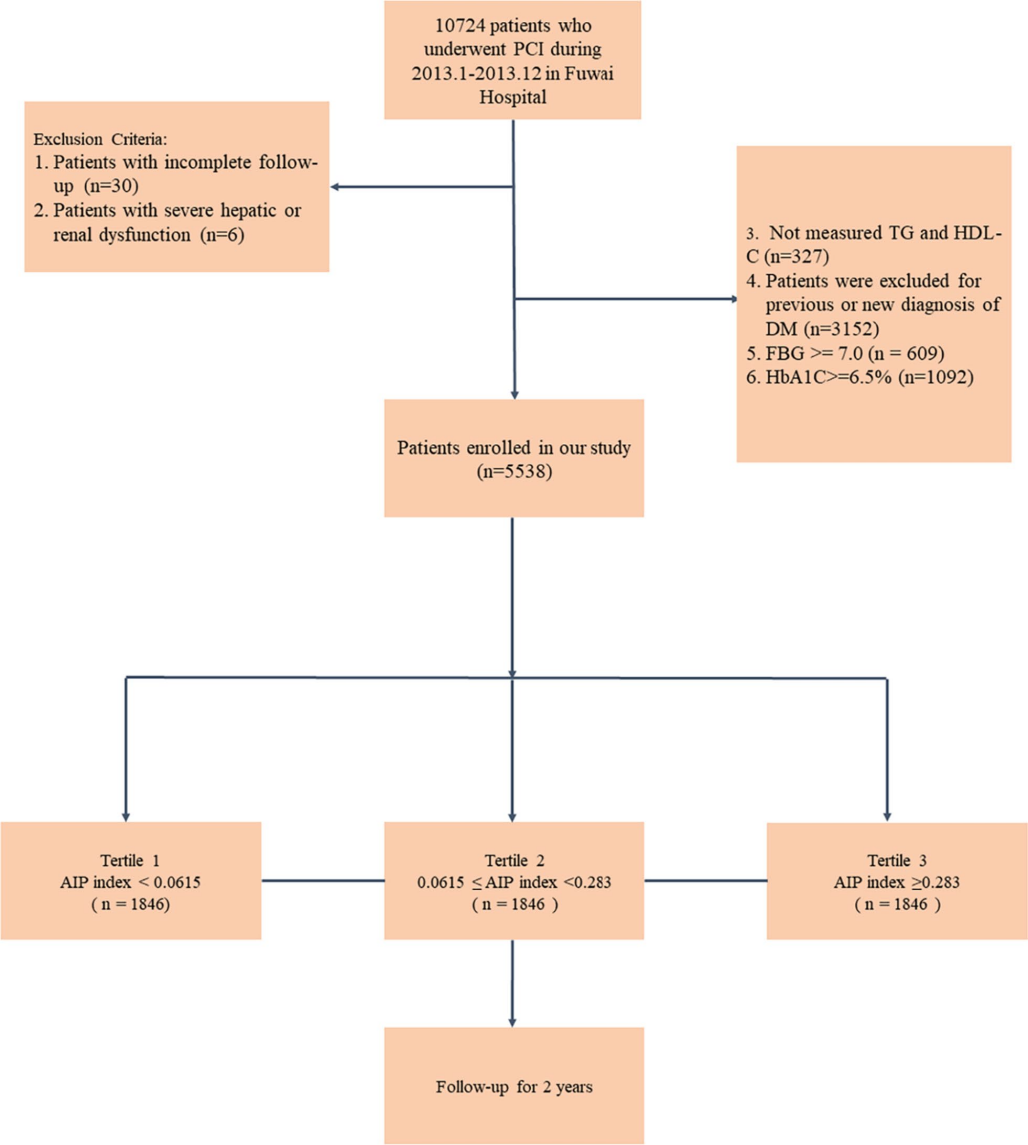
Study flowchart. PCI, percutaneous coronary intervention; DM, diabetes mellitus; FBG, fasting blood glucose; HbA1C, hemoglobin A2C; AIP, atherogenic index of plasma; TG, triglyceride; HDL-C, high-density lipoprotein cholesterol

Non-diabetic CAD patients who received PCI in Fuwai Hospital, Chinese National Center for Cardiovascular Diseases, Beijing, were prospectively enrolled from 1st January to 31st December, 2013. Please see Fig. 1 for details. Lab testing and data collection were conducted prior to administering PCI.

Participants were included according to the following: (1) non-diabetic patients underwent PCI; and (2) aged between 18 and 80 years old. Participants were excluded if they did not fulfil the following criteria: (1) Patients with incomplete follow-up; (2) Patients with severe hepatic or renal dysfunction; (3) unmeasured TG or HDL-C; (4) patients with previous or new diagnosis of diabetes; (5) fasting blood glucose ≥ 7.0; and (6) HbA1C ≥ 6.5%.

Ethical approval was obtained through Fuwai Hospital Research Ethics Committee (No. 2013-449). Once the Institutional Review Board approved the protocol and all patients had been provided with information regarding the study had provided informed consent, the study commenced.

### Participants

All non-diabetic CAD participants received PCI, although the procedural method was dependant on specific operators. Participants who had allergies to tracers and other conditions were excluded, given that they could not receive PCI. Participants were further excluded when considering the possibility of underlying diabetes. For those with severe hepatic/renal dysfunction [20, 21] i.e., serum bilirubin ≥ 5 mg/dL (85 mmol/L) and coagulopathy (INR ≥ 1.5 or prothrombin activity < 40%) or a Glomerular Filtration Rate (GFR) of less than 30 mL/min/1.73m^2^, were thought to have a severe lipid profile metabolism dysfunction condition. Therefore, the AIP index was not considered to be used to predict their prognosis in this study. See Fig. 1 for a schematic of the selection process.

Patient characteristics, as well as information around co-existing conditions and procedural details were collected independently by at least two physicians. Patient characteristics, such as hypertension, diabetes, smoker and renal dysfunction, were assessed according to traditional definitions described in CAD patients guidelines. This meant that the traditional definition of controlled hypertension was established using an SBP ≥ 140 mmHg or a DBP ≥ 90 mm Hg, and/or the current use of antihypertensive medication. Type 2 diabetes mellitus was also defined as those with a a fasting plasma glucose ≥ 126 mg/dL; a 2-h oral glucose tolerance test value ≥ 200 mg/dL; hemoglobin A1C ≥ 6.5%; or random plasma glucose ≥ 200 mg/dL with classic symptoms of hyperglycemia. Smoking status was defined around those who continued to smoke and those who had smoked more than 100 cigarettes. Renal dysfunction was determined in those with a GFR less than 90 mL/min per 1.73 m^2^. Dyslipidemia was diagnosed when patients had low-density lipoprotein cholesterol levels of ≥ 140 mg/dL, a high-density lipoprotein cholesterol level of < 40 mg/dL, or a triglyceride level of ≥ 150 mg/dL [22–24]. Participants were then assigned to one of three groups according to AIP index levels. Each AIP index score was calculated using a base 10 logarithm with a ratio of triglyceride concentrations to high-density lipoprotein-cholesterol. Tertiles were defined as follow: First group involved participants with an AIP < 0.0615, the second tertile with an AIP ≥ 0.0615 but less than 0.283. The third tertile had an AIP of ≥ 0.283. CAD diagnoses were also standardized and based on coronary angiography.

### Treatments

Before receiving selective PCI, patients who had not taken long-term aspirin and P2Y12 inhibitor, received 300 mg aspirin and a P2Y12 inhibitor with a loading dose. Patients who were scheduled for primary PCI received a same dose of aspirin and Clopidogrel with a loading dose 300 mg or 600 mg, according to bleeding risk.

During PCI, 50–100 U/kg of heparin sodium was administered according bleeding risk. From the vision of CAG, more than 50% stenosis of the left main artery (LM), left anterior descending artery (LAD), left circumflex artery (LCX), right coronary artery (RCA), and the main branch of these vessels were defined as coronary artery stenosis. Patients with greater than 70% stenosis in vessels and ischemic symptoms were recommended for coronary stent implantation.

### Follow-up

Participants were visited at 30 days, 6 months, and each year thereafter PCI. Follow-up was considered complete at the 2 years juncture. Primary endpoints were MACEs, consisting of cardiac death, target vessel revascularization (TVR), and non-fatal myocardial infarction (MI). The definition for non-fatal myocardial infarction in this study was defined as Type I myocardial infarction after PCI. Cardiac death was defined as any death which did not have a clear extracardiac origin, or myocardial infarction in accordance with guidelines [25]. Target vessel revascularization was defined as angina or ischemia referable to the target vessel requiring repeat PCI or Coronay Artery Bypass Graft, otherwise known as CABG [26]. Secondary endpoints included components of MACEs, composite of cardiac death and MI, all-cause death, and stroke. Outpatient follow-up was completed by cardiologists in Fuwai Hospital. For those unable to return to the hospital, follow-up was completed over the telephone by clinical workers and clinical research coordinators.

### Statistical analysis

This prospective observational study was designed to determine whether AIP index could predict the long-term prognosis of non-diabetic patients after PCI. Continuous variables are presented as means with corresponding standard deviations (SD), while categorical data are presented as counts with percentages.

Mann–Whitney’s U-test and Student’s T-tests were implemented to compare continuous variables. Standard Chi-square tests were used to compare categorical variables. Cumulative event rates were generated using Kaplan–Meier curves and two-sided Log-rank tests. In instances where the assumptions of proportional hazard for survival analysis were met, Cox’s survival analysis was considered to control confounding factors.

Univariate regression analysis and multivariate analysis were carried out to identify possible predictors for non-diabetic patients after PCI. The sample size made it possible to investigate all predictors through univariate and multivariate models. Restricted cubic spline analysis was conducted to explore correlations between the AIP index and. hazard ratios (HR). HRs with corresponding 95% confidence intervals (CI) were calculated using the Mantel-Cox method. A *p* value of less than 0.05 was considered statistically significant.

Further subgroup analysis was conducted to explore consistency among important subgroups in non-diabetic participants in terms of clinical significance. The p for interactions was measured to explore interactions between AIP index levels and subgroups. p values lower than 0.05 were again considered statistically significant.

All statistical analyses were performed by two investigators (YZ, JY at Fuwai, Hospital) with guidance from a clinical epidemiologist (SS). SPSS (version 26.0), Graphpad Prism (version 8.0), and Rstudio (Rversion 4.0) were used to perform all statistical analyses.

## Results

### Baseline characteristics

Between January and December, 2013, 5538 consecutive CAD participants who had received PCI were enrolled. The mean duration of follow-up was 28 months (SD = 2.3) for patients after the first PCI. As has been described, participants were assigned to one of three AIP groups i.e. the first tertile (n = 1846); second tertile (n = 1846), and the third tertile (n = 1846). Please see Table 1 for further details.

**Table 1:** Baseline characteristics according to different AIP index groups

Variables	Tertile 1 (n = 1846)	Tertile 2 (n = 1846)	Tertile 3 (n = 1846)	P value
Demographic characteristics				
AIP index	− 0.10 ± 0.13	0.17 ± 0.06	0.46 ± 0.15	< 0.001*
Age, years	60.55 ± 9.94	57.33 ± 10.10	54.35 ± 10.31	< 0.001*
BMI, kg/m2	24.80 ± 3.36	25.67 ± 3.00	26.47 ± 2.97	< 0.001*
Male, %	1366 (74)	1463 (79.3)	1565 (84.8)	< 0.001*
CAD, %				
CCS	703 (38.1)	722 (39.1)	681 (36.9)	0.38
ACS	1143 (61.9)	1124 (60.9)	1165 (63.1)	
Coexisting conditions, %				
Hypertension	1156 (62.6)	1092 (59.2)	1110 (60.1)	0.084
Hyperlipidemia	1077 (58.3)	1140 (61.8)	1299 (70.4)	< 0.001*
Renal dysfunction	669 (36.2)	691 (37.4)	634 (34.3)	0.143
Smoker	960 (52.1)	1059 (57.5)	1207 (65.5)	< 0.001*
Cerebrovascular diseases	187 (10.1)	169 (9.2)	140 (7.6)	0.024*
COPD	49 (2.7)	40 (2.2)	33 (1.8)	0.198
Previous myocardial infarction	484 (26.2)	660 (35.8)	723 (39.2)	< 0.001*
Previous CABG	63 (3.4)	65 (3.5)	64(3.5)	0.984
Previous PCI	393 (21.3)	398 (21.6)	374(20.3)	0.593
Peripheral vascular diseases	40 (2.2)	33 (1.8)	35(1.9)	0.692
Lab test				
TG, mmol/l	1.03 ± 0.26	1.51 ± 0.33	2.56 ± 1.13	< 0.001*
TC, mmol/l	4.05 ± 0.99	4.14 ± 1.03	4.42 ± 1.12	< 0.001*
Creatine, umol/l	73.14 ± 14.10	75.75 ± 14.55	76.79 ± 14.80	< 0.001*
Urine acid, umol/l	321.46 ± 73.58	351.23 ± 79.93	377.06 ± 84.63	< 0.001*
HbA1C, %	5.88 ± 0.33	5.88 ± 0.34	5.88 ± 0.33	0.216
HDL-C, mmol/l	1.28 ± 0.28	1.02 ± 0.20	0.86 ± 0.18	< 0.001*
LDL-C, mmol/l	2.37 ± 0.87	2.54 ± 0.89	2.63 ± 0.94	< 0.001*
Albumin, g/l	43.04 ± 4.17	42.92 ± 4.00	43.06 ± 4.03	0.542
LVEF, %	64.03 ± 6.62	63.19 ± 7.32	62.74 ± 7.22	0.003*
Angiographic and procedural details				
Left main involved, %	104 (5.6)	119 (6.4)	89 (4.8)	0.101
CTO	138 (7.5)	144 (7.8)	181 (9.8)	0.022*
Number of stents	1.72 ± 1.00	1.79 ± 1.05	1.86 ± 1.10	0.218
Diameters of stents	3.03 ± 0.53	3.03 ± 0.51	3.05 ± 0.53	0.259
SYNTAX score	11.12 ± 7.47	11.79 ± 7.66	11.91 ± 7.72	0.003*
TVD, %	687 (37.2)	737 (39.9)	731 (39.6)	0.183
Medicine at discharge				
β-blocker	1475 (82.4)	1563 (87.6)	1576 (88.0)	0.001*
Statin	1732 (96.8)	1730 (97.0)	1717 (95.9)	0.19
ACEI/ARB	902 (50.4)	958 (53.7)	988 (55.2)	0.012*
DAPT	1833 (99.3)	1828 (99.0)	1831 (99.2)	0.782
CCB	884 (49.4)	825 (46.3)	804 (44.9)	0.022*

AIP index, atherogenic index in plasma; BMI, body mass index; COPD, chronic obstructive pulmonary disease; CAD, coronary artery disease; CABG, coronary artery bypass graft; PCI, percutaneous coronary intervention; TG, triglycerides; TC, total cholesterol; HDL-C, high-density lipoprotein cholesterol; HbA1C, hemoglobin A2C; LDL-C, low-density lipoprotein cholesterol; LVEF, left ventricular eject fraction; LAD, left anterior descending; RCA, right coronary artery; LCX, left circumflex; TVD, three vessel disease; CTO, chronic total occlusion; SYNTAX, synergy between PCI with TAXUS™ and cardiac surgery; ACEI, angiotensin converting enzyme inhibitors; DAPT, dual antiplatelet therapy; CCB, calcium channel blocker

^*^p < 0.05

Patients in the high AIP index group were younger than those in the low AIP index group (*p* < 0.001), with increased prevalence of men (84.8% vs 79.3% vs 74%, *p* < 0.001), a higher overall BMI (26.47 vs 25.67 vs 24.80, *p* < 0.001), and higher prevalence of hyperlipidemia (70.4% vs 61.8% vs 58.3%, *p* < 0.001). There were also more smokers (65.5% vs 57.5% vs 52.1%, *p* < 0.001), and more previous myocardial infarction (39.2% vs 35.8% vs 26.2%, *p* < 0.001) in the highest AIP index group. The high AIP index group also encountered significantly more metabolic dysfunctions, with increased TG, TC, Creatine, Urine acid, LDL-C, and decreased HDL-C (All *p* < 0.001). In terms of angiographic details, the high AIP index group had a higher incidence of CTO lesions (9.8% vs 7.8% vs 7.5%, *p* = 0.022).

### Univariate, multivariate, and colinearity analysis

Colinearity analysis was conducted to identify correlations between the AIP index and clinical characteristics. As seen in Table 2, we found that TC and LDL-C had a high correlation with the AIP index, of which variance inflation factors (VIFs) were all greater than 10. In addition, there were correlations between TG, HDL-C and the AIP index, with VIFs of 4.494 and 3.452, respectively. These strong correlational values were excluded as further confounding factors used for adjustments.

**Table 2:** Co-linearity analysis of baseline variables and AIP index

Variables	Unstandardized coefficients		Standardized coefficients	t	Sig.	Collinearity statistics	
	B	Std.error	Beta			Tolerance	VIF
(Constant)	0.077	0.046		1.665	0.096		
Demographic characteristics							
Age	< 0.001	< 0.001	− 0.009	− 1.769	0.077	0.648	1.542
BMI	0.002	< 0.001	0.02	4.286	< 0.001	0.864	1.157
Male	0.037	0.004	0.058	9.126	< 0.001	0.445	2.249
CCS	0.002	0.002	0.003	0.76	0.447	0.935	1.069
Coexisting conditions, %							
Hypertension	< 0.001	0.003	− 0.001	− 0.146	0.884	0.746	1.341
Dyslipidemia	0.01	0.002	0.019	4.261	< 0.001	0.947	1.056
Renal dysfunction	0.008	0.004	0.014	2.176	0.03	0.414	2.417
Smoker	0.003	0.003	0.005	0.996	0.319	0.716	1.398
Cerebrovascular diseases	− 0.004	0.004	− 0.004	− 0.891	0.373	0.953	1.049
COPD	− 0.003	0.007	− 0.002	− 0.402	0.688	0.973	1.027
Previous myocardial infarction	0.011	0.003	0.02	3.91	< 0.001	0.687	1.456
Previous CABG	0.004	0.009	0.002	0.48	0.631	0.921	1.086
Previous PCI	− 0.007	0.003	− 0.011	− 2.44	0.015	0.852	1.174
Peripheral vascular diseases	− 0.018	0.008	− 0.01	− 2.293	0.022	0.98	1.02
Lab test							
TG, mmol/l	0.197	0.002	0.732	81.299	< 0.001	0.223	4.494
TC, mmol/l	− 0.059	0.006	− 0.24	− 9.065	< 0.001	0.026	38.825
Creatine, umol/l	< 0.001	< 0.001	0.022	2.987	0.003	0.334	2.995
Urine acid, umol/l	< 0.001	< 0.001	0.023	4.523	< 0.001	0.706	1.417
HbA1C, %	0.008	0.003	0.011	2.421	0.015	0.921	1.086
HDL-C, mmol/l	− 0.417	0.007	− 0.459	− 58.223	< 0.001	0.29	3.452
LDL-C, mmol/l	0.077	0.007	0.269	11.467	< 0.001	0.033	30.482
Albumin, g/l	0.002	< 0.001	0.029	6.001	< 0.001	0.757	1.321
LVEF, %	< 0.001	< 0.001	< 0.001	0.018	0.986	0.81	1.235
Angiographic and procedural details							
Left main involved, %	0.006	0.005	0.005	1.128	0.259	0.854	1.171
CTO	0.011	0.005	0.011	2.364	0.018	0.908	1.101
Number of stents	− 0.001	0.001	− 0.004	− 0.887	0.375	0.769	1.3
Diameters of stents	0.002	0.002	0.003	0.727	0.467	0.877	1.14
SYNTAX score	< 0.001	< 0.001	0.006	1.087	0.277	0.666	1.502
TVD, %	− 0.001	0.002	− 0.003	− 0.551	0.582	0.827	1.209
Medicine at discharge							
β-blocker	0.003	0.003	0.003	0.798	0.425	0.975	1.026
Statin	− 0.013	0.006	− 0.009	− 2.196	0.028	0.988	1.012
ACEI/ARB	0.003	0.002	0.005	1.119	0.263	0.796	1.256
DAPT	− 0.006	0.009	− 0.003	− 0.648	0.517	0.99	1.011
CCB	− 0.002	0.002	− 0.004	− 0.828	0.408	0.83	1.205

AIP index, atherogenic index of plasma; BMI, body mass index; COPD, chronic obstructive pulmonary disease; CAD, coronary artery disease; CABG, coronary artery bypass graft; PCI, percutaneous coronary intervention; TG, triglycerides; TC, total cholesterol; HDL-C, high-density lipoprotein cholesterol; HbA1C, hemoglobin A2C; LDL-C, low-density lipoprotein cholesterol; LVEF, left ventricular eject fraction; LAD, left anterior descending; RCA, right coronary artery; LCX, left circumflex; TVD, three vessel disease; CTO, chronic total occlusion; SYNTAX, synergy between PCI with TAXUSTM and cardiac surgery; ACEI, angiotensin converting enzyme inhibitors; ARB, angiotensin receptor blocker; DAPT, dual antiplatelet therapy; CCB, calcium channel blocker

Univariate and multivariate analyses were performed to determine the predictive value of the AIP index for MACEs in non-diabetic patients underwent PCI at 2 years. As can be seen in Table 3, the AIP index appears to be an independent factor for predicting MACEs in non-diabetic patients after PCI at 2 years (tertile 3 vs. tertile 2: HR: 1.36, 95% CI 1.01–1.82; *p* = 0.042).

**Table 3:** Univariate and multivariate analysis for AIP index predicting the occurrence of MACEs within 2 years after PCI

Variables	Univariate analysis			Multivariate analysis		
	HR	95%CI	P value	HR	95%CI	P value
AIP index						
Tertile 1	Ref	Ref	Ref	Ref	Ref	Ref
Tertile 2	0.96	0.71–1.30	0.801	0.93	0.69–1.27	0.656
Tertile 3	1.37	1.04–1.81	0.025*	1.36	1.01–1.82	0.042*
Age	1	0.99–1.01	0.95	1.00	0.99–1.01	0.849
BMI	1.02	0.98–1.05	0.41	1.05	0.97–1.05	0.687
Male	0.94	0.70–1.25	0.66	0.89	0.59–1.34	0.567
CCS	1.08	0.85–1.36	0.551	1.05	0.82–1.34	0.722
Hypertension	1.02	0.80–1.29	0.894	0.97	0.74–1.28	0.852
Hyperlipidemia	1.2	0.94–1.53	0.152	1.10	0.86–1.42	0.448
Renal dysfunction	1	0.79–1.27	0.993	0.91	0.63–1.32	0.632
Smoker	1	0.79–1.26	0.986	0.92	0.70–1.21	0.551
Cerebrovascular diseases	1.31	0.91–1.88	0.145	1.24	0.86–1.80	0.255
COPD	0.76	0.31–1.84	0.542	0.75	0.31–1.83	0.522
Previous myocardial infarction	1.24	0.98–1.57	0.075	1.06	0.81–1.38	0.676
Previous CABG	1.76	1.08–2.87	0.024*	1.35	0.81–2.26	0.253
Previous PCI	1.63	1.27–2.10	< 0.001*	1.50	1.15–1.95	0.003*
Peripheral vascular diseases	2.69	1.52–4.45	< 0.001*	2.60	1.50–4.49	0.001*
Creatine, umol/l	1	0.99–1.01	0.869	0.99	0.98–1.01	0.321
Albumin, g/l	0.99	0.96–1.01	0.305	0.99	0.96–1.02	0.47
Left main involved, %	1.03	0.63–1.69	0.897	0.88	0.53–1.45	0.611
CTO	2.37	1.75–3.21	< 0.001*	2.14	1.56–2.93	< 0.001*
TVD	1.75	1.39–2.20	< 0.001*	1.68	1.38–2.04	< 0.001*
β-blocker at discharge	1.11	0.78–1.56	0.569	1.04	0.74–1.48	0.81
Statin at discharge	0.59	0.36–0.98	0.042*	0.57	0.35–0.95	0.031*
ACEI/ARB at discharge	1.06	0.84–1.33	0.649	1.00	0.77–1.29	1
CCB at discharge	0.82	0.65–1.04	0.094	0.84	0.65–1.08	0.163

AIP index, atherogenic index in plasma; BMI, body mass index; COPD, chronic obstructive pulmonary disease; CCS, chronic coronary syndrome; CABG, coronary artery bypass graft; PCI, percutaneous coronary intervention; ACEI, angiotensin converting enzyme inhibitors; DAPT, dual antiplatelet therapy; CCB, calcium channel blocker

^*^p < 0.05

### Primary and secondary endpoints

**Fig. 2:**
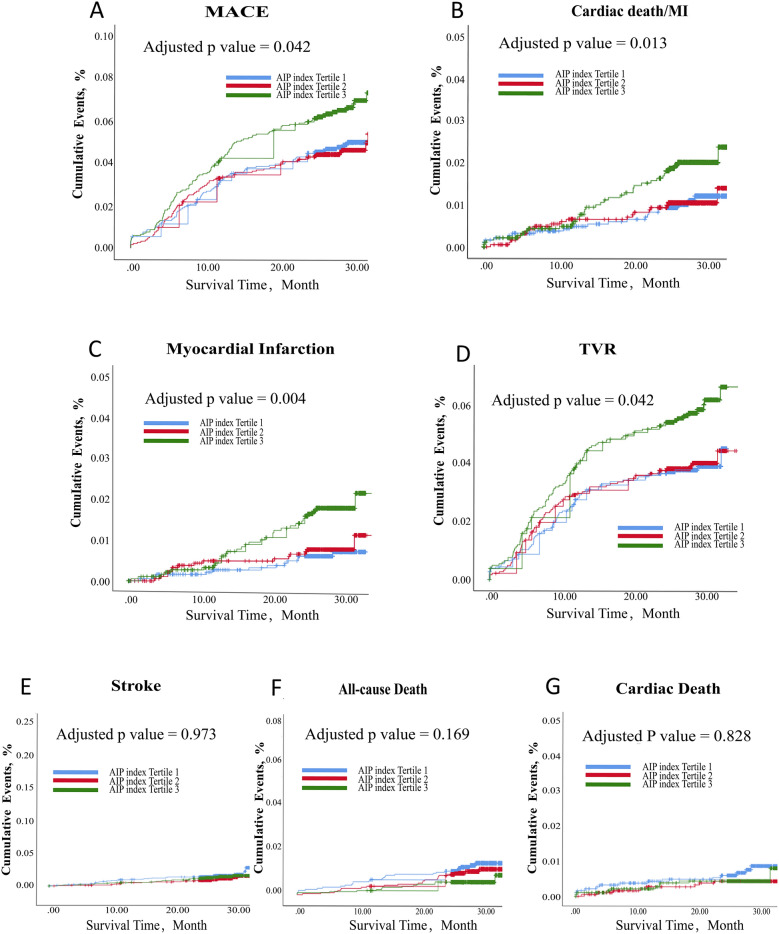
Kaplan–Meier curves according to endpoints in various group

As shown in Fig. 2A, the Kaplan-Meiers curves for the third AIP index tertile highlights a clear increase in MACEs compared to both the first and second tertiles at 2 years after PCI (adjusted *p* = 0.042). Table 4 provides HRs for the first tertile compared with the second and third. The MACE HR at 2 years in the third tertile was higher than in the first (6.5% vs 4.8%; HR: 1.37, 95% CI 1.04–1.81; *p* = 0.025). However, no statistically significant difference was observed between the first and second tertiles. Please see Table 4. For further details.

**Table 4:** Long-term Outcomes According to AIP index before and after Multivariate COX Regression Adjustment

Endpoints	No. of events (%)	Hazard ratio (95% confidence interval)	P value	Adjusted hazard ratio (95% confidence interval)	Adjusted P value
Primary endpoint					
MACE					
Tertile 1	88 (4.8)	Ref	Ref	Ref	Ref
Tertile 2	85 (4.6)	0.96 (0.71–1.30)	0.801	0.93 (0.69–1.27)	0.656
Tertile 3	120 (6.5)	1.37 (1.04–1.81)	0.025*	1.36 (1.01–1.82)	0.042*
Secondary endpoint					
Cardiac death/MI					
Tertile 1	20 (1.1)	Ref	Ref	Ref	Ref
Tertile 2	21 (1.1)	1.03 (0.56–1.91)	0.919	1.10 (0.59–2.06)	0.766
Tertile 3	36 (2.0)	1.81 (1.05–3.13)	0.033*	2.10 (1.17–3.78)	0.013*
All-cause death					
Tertile 1	27 (1.5)	Ref	Ref	Ref	Ref
Tertile 2	23 (1.2)	0.83 (0.48–1.46)	0.521	1.00 (0.55–1.80)	0.993
Tertile 3	13 (0.7)	0.48 (0.25–0.93)	0.029*	0.60 (0.29–1.24)	0.169
Cardiac death					
Tertile 1	14 (0.7)	Ref	Ref	Ref	Ref
Tertile 2	9 (0.5)	0.61 (0.27–1.42)	0.256	0.88 (0.37–2.13)	0.777
Tertile 3	9 (0.5)	0.64 (0.28–1.47)	0.289	1.10 (0.45–2.71)	0.828
TVR					
Tertile 1	70 (3.8)	Ref	Ref	Ref	Ref
Tertile 2	72 (3.9)	1.03 (0.74–1.43)	0.866	0.98 (0.70–1.37)	0.903
Tertile 3	105 (5.7)	1.51 (1.11–2.03)	0.008*	1.40 (1.01–1.93)	0.042*
Stroke					
Tertile 1	32 (1.7)	Ref	Ref	Ref	Ref
Tertile 2	23 (1.2)	0.69 (0.40–1.18)	0.176	0.75 (0.42–1.33)	0.319
Tertile 3	27 (1.5)	0.83 (0.50–1.39)	0.478	1.01 (0.58–1.75)	0.973
Myocardial infarction					
Tertile 1	12 (0.7)	Ref	Ref	Ref	Ref
Tertile 2	15 (0.8)	1.26 (0.59–2.67)	0.564	1.23 (0.57–2.66)	0.605
Tertile 3	32 (1.7)	2.69 (1.38–5.22)	0.004*	2.82 (1.39–5.72)	0.004*

Confounding factors for adjustment: common and possible confounding factors described in Table 3 were enrolled, including age, male, BMI, classification of CAD, hypertension, hyperlipidemia, renal dysfunction, smoker, cerebrovascular diseases, pre-PCI, pre-CABG, pre-myocardial infarction, COPD, peripheral vascular disease, CTO, TVD, creatine, albumin, left main involved, β-blocker at discharge, statin at discharge, ACEI/ARB at discharge, CCB at discharge

MACE, major adverse cardiovascular events; COPD, chronic obstructive pulmonary disease; PCI, percutaneous coronary intervention

^*^p < 0.05

Figures 2B–G show secondary endpoints of different AIP tertiles with Kaplan–Meier curves. As can be seen, TVR appears to be the main driver behind the increase of MACEs across different groups (adjusted *p* = 0.042). HRs for TVR at 2 years in the third tertile was higher than that in the first (5.7% vs 3.8%; HR: 1.51, 95% CI 1.11–2.03; *p* = 0.008). The incidence rate of MI and composite endpoint of MI and cardiac death also increased in tertile 3 although with low incidence rate (Cardiac death/MI: adjusted *p* = 0.013; MI: adjusted *p* = 0.004). HR for cardiac death/MI and MI at 2 years in the third tertile was higher than that in the first (cardiac death/MI: 2.0% vs 1.1%; HR: 1.81, 95% CI 1.05–3.13, *p* = 0.033; MI: 1.7% vs. 0.7%; HR: 2.69, 95% CI 1.38–5.22, *p* = 0.004). No other endpoints differed significantly among the groups at 2 years. Please see Table 4. For further details.

#### Restricted cubic spline and subgroup analysis

**Fig. 3:**
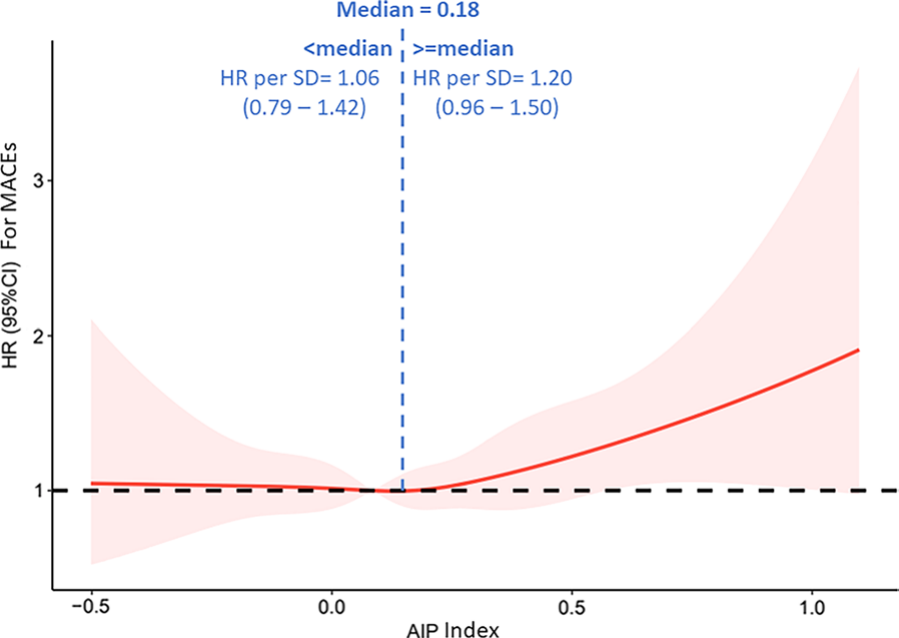
Restricted cubic spline of MACEs and AIP index. HR, hazard ratio; SD, standard derivation; AIP, atherogenic index of plasma

There does not appear to be a parallel relationship in Kaplan-Meiers curves between tertile one and two. Further restricted cubic spline (RCS) analysis was conducted to identify correlations between the HR for MACEs and the AIP index. As shown in Fig. 3, the RCS curve appears to be a J-shaped with the HR for MACEs significantly increasing as the AIP index increased over and above 0.18. For AIP index less than 0.18, the HR per SD was 1.06, with the 95% CI ranging from 0.79 to 1.42. However, when AIP index was over 0.18, the HR per SD was 1.20, with 95% CI ranging from 0.96 to 1.50.

**Fig. 4:**
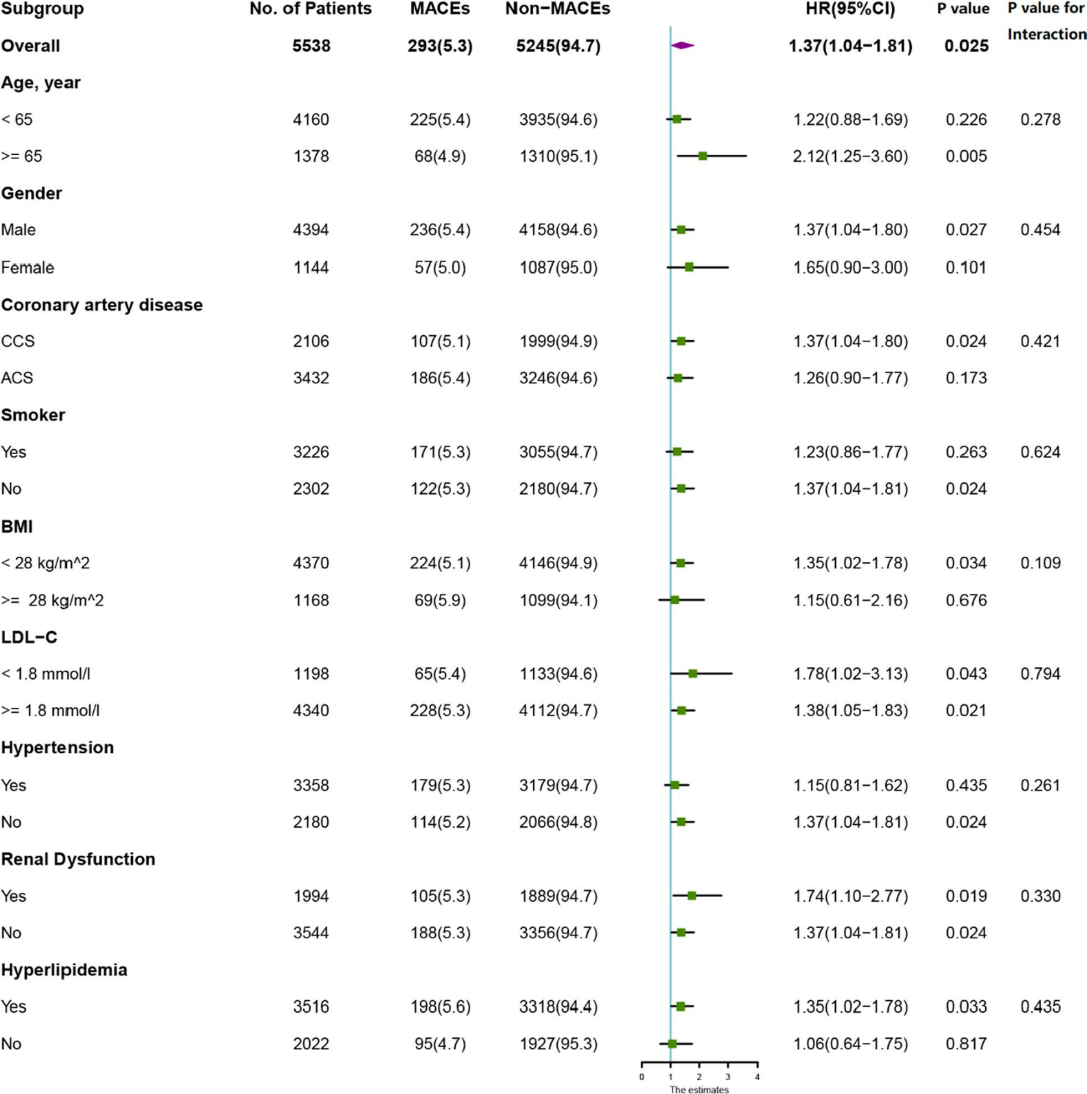
Subgroup analysis according to High AIP index refer to low AIP index. MACEs, major adverse cardiovascular events; CCS, chronic coronary syndrome; ACS, acute coronary syndrome; BMI, body mass index; LDL-C, low-density lipoprotein cholesterol; CI, confidence interval

Further subgroup analysis was conducted to explore consistency among the significant subgroups. As can be seen in Fig. 4, all HRs in different subgroups were over 1, including the gender, age, obesity, classification of CAD, hypertension, high or low LDL-C groups, and the renal dysfunction group. However, only *p* values in aged, in men, non-smokers, with normal BMI, in the CCS group, high or low LDL-C groups, with or without renal dysfunction groups, the non-hypertensive group, and hyperlipidemia group were less than 0.05. No interaction between subgroups and AIP index was observed (all *p* for interaction > 0.05).

## Discussion

This study focused on using the standard AIP index for non-diabetic CAD patients after PCI. The aim was to develop evidence-based insights for clinical practice. We found that high AIP index levels are closely related to a higher incidence rate of MACEs after PCI in non-diabetic patients. We also found a relationship between the AIP index and MACE hazard ratios in the form a J-shaped curve. The significant increase appears to occur when the AIP index increases beyond 0.18. This evidences the potential impact of metabolic dysfunction on prognosis for non-diabetic patients after PCI therefore, the AIP index could be used to predict outcomes. There is also the possibility to combine the AIP with other factors which would provide an even more accurate, reliable prognostic tool.

The AIP index has already been established as having value for assessing metabolic dysfunction in non-diabetic patients. As reported by Nordestgaard et al. [27] in 2016, high TG positively correlates with a higher incidence rate of atherosclerosis plaque, and therefore is associated with poorer prognosis. As traditional lipid particle analysis indicates, higher HDL-C within the normal range suggests there is an opposing effect of triglyceride on atherosclerosis, higher HDL-C has a protective effect and higher triglyceride has a detrimental role on atherosclerosis [3, 28]. Whereas, a lower HDL-C level appears to inhibit anti-atherogenic properties and the anti-oxidation effect. These particularly interesting effects appear to be due to diminishing HDL-C levels which are usually observed prior to the appearance of glycemic dysregulation [29]. Through a previous study, we found that a higher trigylceride glucose index score does not accurately represent a decrease in HDL-C [18]. The explains why the TyG index does not appear suitable for non-diabetic patients and why the AIP index may prove useful.

This study does suggest that the AIP index can be used to accurately predict outcomes for non-diabetic patients after PCI. We found that those with a high AIP index contributed almost 30 percent of the increase in MACEs when compared to those with a low AIP index. MACEs appear to be mainly driven by target vessel revascularization at 2 years, which appears consistent with the characteristics of the AIP index. As Yang et al. [18] reported, the TyG index does not accurately predict prognosis for non-diabetic CAD patients. Although, HbA1C has been used for non-diabetic patient prognosis with varying degrees of accuracy [30–32]. Which suggests there is a need to intercalate one (or more) inflammation markers into a cohesive nomogram or with larger datasets into an artificial intelligence model. This is a growing body of evidence however, currently ADMA, ANGPTL2, MMP-9, and hs-CRP [33, 34] have been investigated as show a degree of promise. Despite these strides forward, there remains a number of problems with the established indices. Therefore, we cannot say with certainty that these indices are useful for non-diabetic patients with signs of metabolic dysfunction. This knowledge base is especially vague for those at high risk of metabolic syndrome before the developing hyperglycemia. Therefore, the AIP index provides a number of advantages over previous indices. The AIP could be used for predicting the occurrence of ischemic heart diseases, and also for predicting the dynamic progression of atherogenic disease, especially for those without obvious changes in blood glucose.

Interestingly, the correlation between the AIP index, taken as a continuous measure, in relation to the hazard ratio for MACEs for non-diabetic patients after PCI appeared J-shaped. While this is a relatively common occurrence [35], it is important because this can highlight a threshold of sorts. In this instance, we can say that there does not appear to be a protective effect when patients have an AIP index of lower than 0.18. An explanation for this may be the effect of HDL-C, as has been described. Previous studies have also found a U-shaped association which has further fueled the discussion on the pathophysiological role of HDL in cardiovascular diseases [36]. This may mean that a single assessment method for CVD risk using HDL-C is unsuitable. However, it may also explain why we found that a very low AIP score does not highlight a preventive effect for MACEs. These findings are consistent with our clinical work, which suggest that a normal AIP index range (less than 0.18) correlates with TG and HDL-C, and does not influence CAD patient prognoses. A J shaped association indicated that when the very low AIP score existed, possible equilibrium may occur between the possible preventive effect of lower TG and the detrimental effect of extremely higher HDL-C. Another explanation is about the various HDL-C functionalities, different subclasses of HDL-C should be concentrated, which may explain the U-shaped curve of HDL-C and J-shaped curve of AIP index to some extent [37–39]. On the other hand, when an AIP index score rises beyond the normal upper range, outcomes are impacted. We found, the higher the AIP index, the more severe the prognosis becomes, which suggests that combining triglycerides with high-density lipoprotein cholesterol measures increase prognostic accuracy for these patients although, there are likely to be differences within any sample of patients.

Participants across different ethnicities also present with heterogeneous symptoms and severities. As reported by Leigh et al. [40], minorities in USA have different outcomes when diagnosed with coronary heart disease. Those of African origin also appear to have a higher incidence of first MI or fatal CHD incidence compared to Caucasian populations [41]. Skowronski et al. [42] also reported that Asians have smaller dimensions of all proximal coronary arteries, most prominently displayed in left main coronary artery (LMCA) when compared with Caucasians. Researchers focusing on Chinese populations tend to use ticagrelor in patients with stable coronary artery disease, and have found that ticagrelor exposure is higher than in Caucasians [43]. Our study focused on a Chinese population, and found that AIP index is a novel index for predicting prognosis in non-diabetic patients after PCI. More research across ethnicities is required because there may be subtle differences which can be used for nomogram development or in artificial intelligence studies.

Subgroup analysis was conducted to further understand some of factors involved and perhaps to identify those most at risk. It is important to note that even though the p-values for interactions did not highlight significant interactions, this may be because of the size of each subgroups sample was relatively small. Additional visual analysis does however suggest that there may be some significant factors. That is to say, being male, a non-smoker, 65 years of age and older, with CCS and having a BMI of less than 28, but without hypertension, may mean a substantially high risk although further research would be needed to verify this claim. What can be said is that this early evidence further recommends nomogram development to explore these interactions further. Subgroup analysis is a necessary step in this type of research however again any recommendations made here are tentative given the limitations.

In combination with related studies predicting prognosis for non-diabetic patients, AIP index is thought to have priority [18, 44]. For non-diabetic patients who had previous received PCI therapy upon admission, an assessment of metabolism dysfunction could be useful. At this time, an AIP index calculation could well document the possible risk of MACEs for out-patients. A higher AIP index value suggests to clinicians that patients are likely to be at high-risk of metabolism dysfunction, which is a serious serum lipid control issue requiring urgent lifestyle adjustments. Pharmacological treatments could be considered to control lipid profiles when necessary. Clear definitions for detecting HDL-C subclasses may help us to develop more effective lower-lipid strategies.

### Strength and limitation

We conducted this study of the AIP index for non-diabetic patients who received PCI, which provides an option for physicians to identify high-risk patients before the appearance of glycemia. However, this was a relatively small study of non-diabetic patients. There maybe differences in this population in terms of the development of type 2 diabetes and in terms of lifestyles which could not be analyzed here. The J-shaped curve perhaps reflects the physiology equilibrium and dynamic pathological changes in non-diabetic patients with potential metabolic dysfunction, but perhaps not. We only enrolled patients from one center in this study and a multicenter study in China may provide insights which this study could not. Second, we could not report the diagnose of metabolic syndrome due to the lack of post-2 h blood glucose, which may have helped us explore correlations between AIP index and the MeTs before the appearance of hyperglycemia.

## Conclusion

AIP index could be seen as the useful predictor of poor prognosis for those non-diabetic patients after PCI, due to ability to highlight metabolic dysfunction. The relationship between HR and AIP index appears J-shaped. 0.18 could be used as the cutoff for the AIP index, specifically when assessing hazard ratios for non-diabetic patients after PCI. More multi-center studies, designed for non-diabetic patients with potential metabolic dysfunction should be conducted to verify the value of AIP index in this context.

## Data Availability

The datasets used and/or analyzed in the study are available from the corresponding author upon reasonable requests.

## Abbreviations

AIP index: Atherogenic index of plasma
MACE: Major adverse cardiac events
BMI: Body mass index
GFR: Glomerular filtration rate
COPD: Chronic obstructive pulmonary disease
CAD: Coronary artery disease
CABG: Coronary artery bypass graft
PCI: Percutaneous coronary intervention
TG: Triglycerides
TC: Total cholesterol
HDL-C: High-density lipoprotein cholesterol
HbA1C: Hemoglobin A2C
LDL-C: Low-density lipoprotein cholesterol
LVEF: Left ventricular eject fraction
LAD: Left anterior descending
RCA: Right coronary artery
LCX: Left circumflex
TVD: Three vessel disease
CTO: Chronic total occlusion
SYNTAX: Synergy between PCI with TAXUSTM and cardiac surgery
ACEI: Angiotensin converting enzyme inhibitors
ARB: Angiotensin receptor blocker
DAPT: Dual antiplatelet therapy
CCB: Calcium channel blocker
